# P-553. The Impact of Six-Months Antiretroviral Drug Dispensing on Viral Load Suppression in 18 Health Facilities in Mozambique From 2019 to 2023

**DOI:** 10.1093/ofid/ofae631.752

**Published:** 2025-01-29

**Authors:** Evangelina Inacio Namburete Bauaze, Helder Macul, Herminio Nhaguiombe, Erica Bila, Irenio Gaspar, Orrin Tiberi, Morais da Cunha

**Affiliations:** PEPFAR Coordination Office in Mozambique, Maputo, Maputo, Mozambique; ICAP, Maputo, Maputo, Mozambique; CDC in Mozambique, Maputo, Maputo, Mozambique; DOD in Mozambique, Maputo, Maputo, Mozambique; Ministry of Health, Maputo, Maputo, Mozambique; Ministry of Health, Maputo, Maputo, Mozambique; Ministry of Health, Maputo, Maputo, Mozambique

## Abstract

**Background:**

In 2019, Mozambique adopted the six-months antiretroviral drug dispensing (6MMD) for all eligible People Living with HIV (PLHIV) subgroups. Patients on 6MMD, pick up their antiretroviral drugs at the clinics every six months. As of June 2023, the model was rolled out to 85 health facilities (HFs) and overall viral load suppression was 93%. We analyzed United States President’s Plan for AIDS Relief (PEPFAR) program monitoring and evaluation data to understand the impact of six-months drug dispensing on viral load suppression.

**Methods:**

At the end of June 2023, we analyzed Viral Load data obtained from electronic patients tracking system (EPTS) an OpenMRS used by PEPFAR to report HIV data nationally in all PEPFAR supported sites in Mozambique. Based on the volume of clients served ( >1000 PLHIV) by each health facility, we selected 9 out of 85 health facilities that implemented six-months antiretroviral drug dispensing and 9 that were not implementing, and we compared the overall percentage of viral load suppression at each HF in June 2019 and June 2023.Viral load was considered suppressed if < 1000 copies/ml in the last consultation for patient enrolled on 6MMD and in the last 6 months for patient not enrolled on 6MMD. All the 18 HFs were PEPFAR supported sites.

**Results:**

As by June 2023, the 18 HFs selected had 126,719 PLHIV on treatment, representing 6% of PLHIV on treatment in the country. Overall Viral load suppression in June 2019 & June 2023 in the 9 HFs with 6MMD ranged from 69 – 72% & 91 – 94% while in the 9 HFs that were not implementing 6MMD ranged from 69 – 74% & 78 – 89%.

**Conclusion:**

In the sites we assessed, six-months antiretroviral drug dispensing contributed to reduce HIV transmission by improving viral load suppression rates in Mozambique.

**Disclosures:**

**All Authors**: No reported disclosures

